# The Aberrant Origin of the Suprascapular Artery May Hide Neural Covariants: A Cadaveric Finding

**DOI:** 10.7759/cureus.44571

**Published:** 2023-09-02

**Authors:** George Tsakotos, George Triantafyllou, Christos Koutserimpas, Vasileios Karampelias, Maria Piagkou

**Affiliations:** 1 Anatomy, National and Kapodistrian University of Athens, Athens, GRC; 2 Orthopaedics and Traumatology, 251 Hellenic Air Force General Hospital, Athens, GRC

**Keywords:** cadaver dissection, interconnection, communication, long thoracic nerve, phrenic nerve, anatomical variation, suprascapular artery

## Abstract

The axillary artery is the continuation of the subclavian artery. Occasionally, some of the subclavian artery’s distal branches may atypically originate from the axillary artery, such as the suprascapular artery. The suprascapular artery’s distal (low) origin from the axillary artery, instead of the subclavian artery, may also be characterized as an aberrant suprascapular artery. The current cadaveric report describes the coexistence of an aberrant suprascapular artery (of axillary origin), variant course, and termination with atypically formatted nerves originating from the cervical (the phrenic nerve) and the brachial (the long thoracic and the median nerves) plexus. An unusual interconnection between the phrenic and the long thoracic nerves was also described. The aberrant suprascapular artery had an atypical termination below the superior transverse scapular ligament, along with the suprascapular vein and nerve. Except for the atypically formatted phrenic and long thoracic nerves, the aberrant suprascapular artery coexisted with an atypical passage of the anterior ramus of the C6 spinal nerve, through the middle scalene muscle, before the long thoracic nerve formation, and a variant formation of the median nerve. Understanding neurovascular variants is crucial for interventionists and surgeons who work in the supra- and infraclavicular areas. Being aware of the different origins of the brachial plexus branches, in the supraclavicular part, may help reduce the occurrence of iatrogenic axillary injury. Efforts should be made to expand the number of cadaveric studies that investigate the origin, course, interconnection, and branching patterns of these nerves and related covariants, in a systematic way, thus unifying their study and comprehension.

## Introduction

The axillary artery is the subclavian artery’s continuation. The axillary artery’s typical branching pattern includes six branches and presents a high variability [1]. Occasionally, some of the subclavian artery’s distal originating branches may emanate from the axillary artery, independently, or by trunks, such as the suprascapular artery (SSA). The axillary origin of the SSA (from the axillary artery’s first, second, or third part) is characterized as an aberrant SSA. Typically, the SSA emanates from the thyrocervical trunk [1], independently or by a common trunk with the transverse cervical artery. The left SSA continues its course, posterior to the clavicle and the subclavius muscle, and crosses the subclavian artery and the brachial plexus (BP) [1]. The SSA is accompanied by the suprascapular nerve (SSN) and commonly passes over the superior transverse scapular ligament, while the suprascapular vein (SSV) and the SSN course beneath the ligament [1]. A cadaveric report by Piagkou et al. [2] emphasized the coexistence of an aberrant SSA of a variant course and termination with variant nerves’ formations.

The current cadaveric report describes the coexistence of an aberrant SSA (of axillary origin, course, and termination) with atypically formatted nerves originating from the cervical plexus (the phrenic nerve) and the BP (the long thoracic nerve (LTN) and the median nerve). An unusual interconnection between the phrenic nerve and the LTN was also described.

## Case presentation

**Figure 1:**
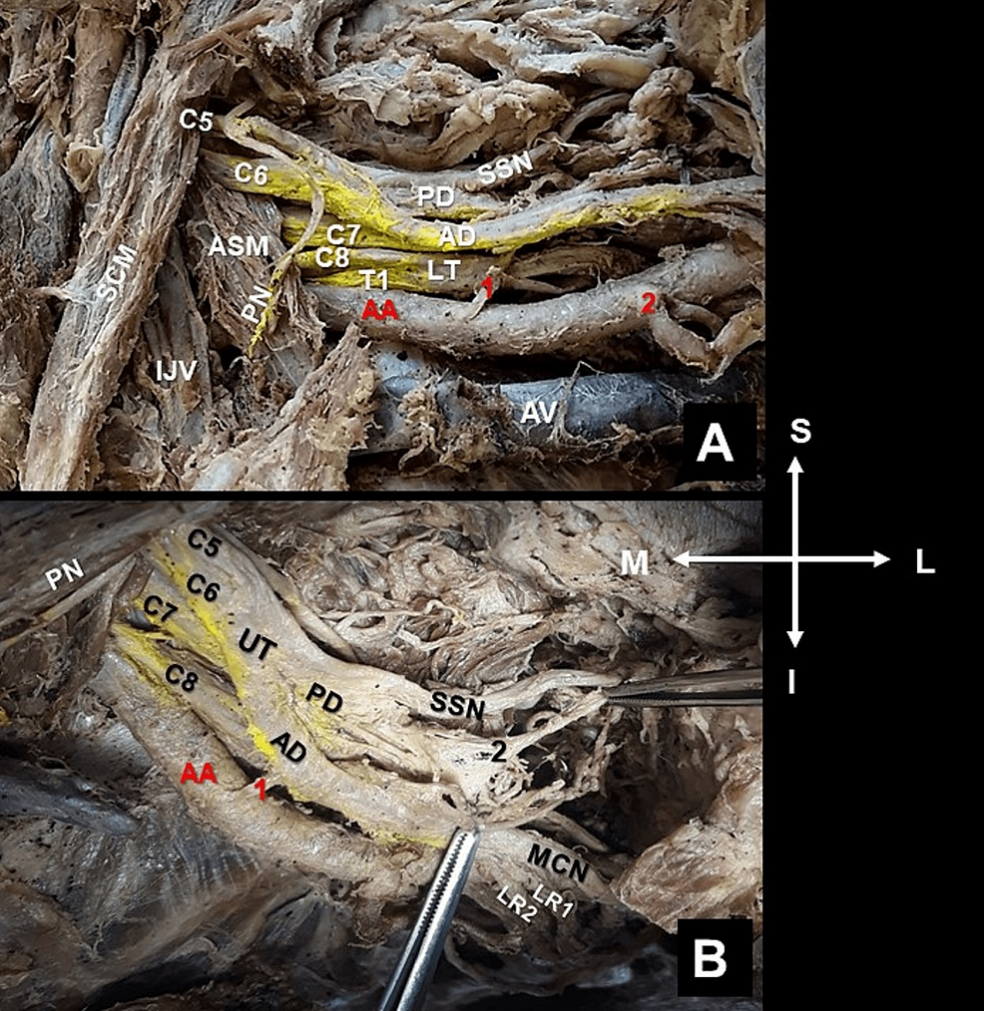
Left-sided supra- and infraclavicular view. The aberrant suprascapular artery (SSA), of axillary origin, and the ipsilateral suprascapular nerve (SSN), of low origin. A. In brachial plexus formation (fusion of C5, C6, C7, C8, and T1 spinal nerves), the aberrant SSA (1) originated from the first part of the axillary artery (AA), at the level of the SSN emersion from the posterior division (PD) of the upper trunk (UT) of the brachial plexus (C5, C6 fusion), sternocleidomastoid muscle (SCM), anterior scalene muscle (ASM), internal jugular vein (IJV), axillary vein (AV), anterior division (AD), and lower trunk (LT). B. The upper subscapular nerve (2) originates proximal to the brachial plexus PD, with the upper subscapular artery supplying the upper part of the subscapularis muscle. The AD branches into the musculocutaneous nerve (MCN), the double lateral roots (LR1 and LR2) of the median nerve, the phrenic nerve (PN), and (2) the thoracoacromial artery. Orientation (M: medial, L: lateral, S: superior, and I: inferior).

**Figure 2:**
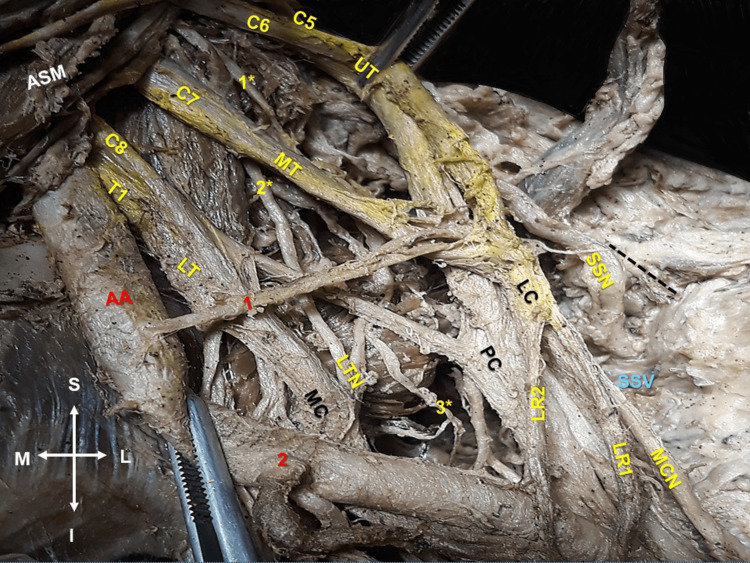
Left supra- and infraclavicular view. Relationship of the aberrant suprascapular artery (1) with the middle trunk’s (MT) and upper trunk’s (UT) anterior and posterior divisions. The aberrant suprascapular artery from the first part of the axillary artery (AA) and its relationship with the MT and the UT anterior and posterior divisions of the brachial plexus (C5, C6, C7, C8, and T1). The formation of the lateral, medial, and posterior cords (LC, MC, and PC). The LC division into the musculocutaneous nerve (MCN), and the double lateral roots (LR1 and LR2) of the median nerve. The emersion of the suprascapular nerve (SSN) from the posterior division of the UT, long thoracic nerve (LTN) formation from the fusion of the C5 and C6 (1*) and C7 (2*) spinal nerves’ roots and the branching pattern (3*) at the upper part of the serratus anterior muscle. (2) Thoracoacromial artery. SSN and suprascapular vein (SSV) passage under the superior transverse scapular ligament (black line depicts the upper border of the scapula, and the ligament is not depicted). The figure orientation (M: medial, L: lateral, S: superior, and I: inferior). ASM: anterior scalene muscle.

During routine dissection of the neck, the supra- and infraclavicular area, and the arm of an 80-year-old formalin-embalmed female donated cadaver of Greek origin, a unilateral (left-sided) aberrant origin of the SSA was identified. The SSA origin, course, and termination patterns were atypical. The aberrant SSA originated from the axillary artery’s first part, at the level of the SSN emersion (Figure 1A). The aberrant SSA coursed through the anterior and posterior division of the C5, C6, and C7 spinal nerves’ fusion (Figures 1B, 2). The SSN atypically originated from the posterior division of the upper trunk (C5-C6 spinal nerves’ fusion) of the BP, was characterized as a low-origin nerve (Figure 1B), and coursed posterior to the axillary artery and the BP. The aberrant SSA bifurcated into a) a branch that coursed below the superior transverse scapular ligament along with the SSN and SSV (Figure 2), and b) a branch (the upper subscapular artery) that supplied the subscapularis muscle’s upper part and was accompanied by the upper subscapular nerve, that emanated from the posterior division of the C5 and C6 spinal nerves’ fusion. The LTN originated from the anterior rami of the C5, C6, and C7 spinal roots.

**Figure 3:**
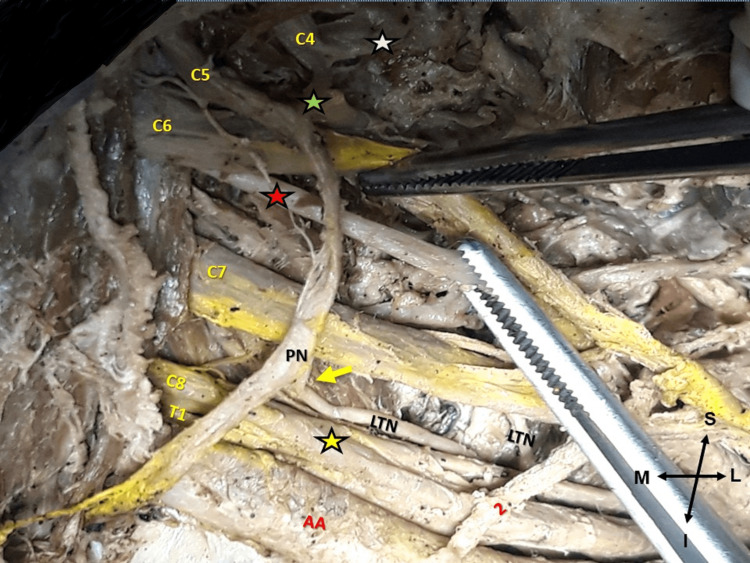
Left supra- and infraclavicular view. The phrenic nerve (PN) and long thoracic nerve (LTN) origins, course, and communication. The PN formation from the contribution of C3 (black-white asterisk), C4 (black-green asterisk), C5, and C6 (black-red asterisk) spinal nerves. The yellow arrow indicates the interconnection of the PN with the LTN and axillary artery (AA), (2) the aberrant suprascapular artery, brachial plexus (C5, C6, C7, C8, T1) branching pattern, and black-yellow asterisk indicates lower trunk. Figure’s orientation (M: medial, L: lateral, S: superior, and I: inferior).

**Figure 4:**
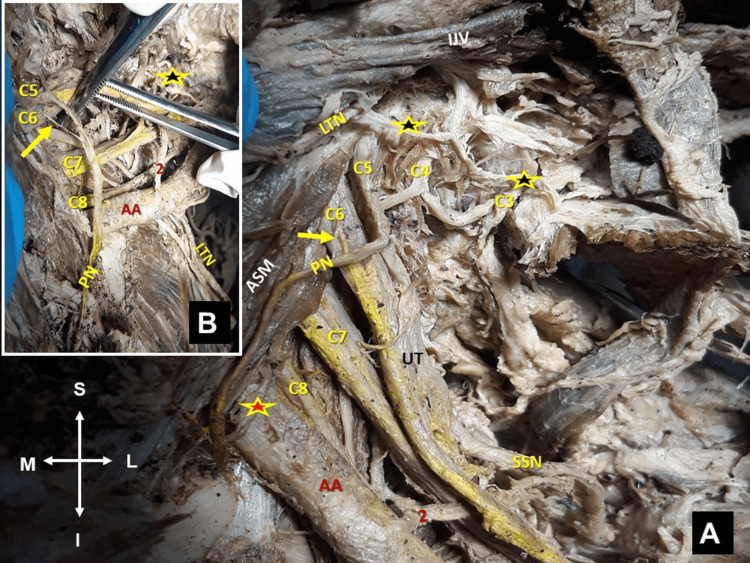
Left supra- and infraclavicular view. The phrenic nerve (PN) formation and the brachial plexus (C5, C6, C7, C8, and T1) branching pattern. (A) The PN formation from the contribution of C3, C4, C5, and C6 (yellow arrow) spinal nerves. (B) Yellow arrow indicates the C6 spinal nerve contribution to the PN; yellow-red asterisk indicates the branch to the deep part of the anterior scalene muscle (ASM), long thoracic nerve (LTN) and its branches to the upper part of the serratus anterior muscle (yellow-black asterisks), internal jugular vein (IJV), and axillary artery (AA), (2) aberrant suprascapular artery, yellow-red asterisk indicates lower trunk. Figure’s orientation (M: medial, L: lateral, S: superior, and I: inferior).

The anterior ramus from the C6 spinal root was identified piercing the middle scalene muscle. At the level of the thoracoacromial artery origin, the LTN received a branch from the phrenic nerve (interconnection of the cervical with the BP) (Figures 3, 4A, 4B). The phrenic nerve originated from the C3, C4, and C5 spinal nerves and received a thin branch from the C6 spinal nerve (resembling an accessory phrenic nerve) and continued its course anterolateral to the anterior scalene muscle (Figures 3, 4).

**Figure 5:**
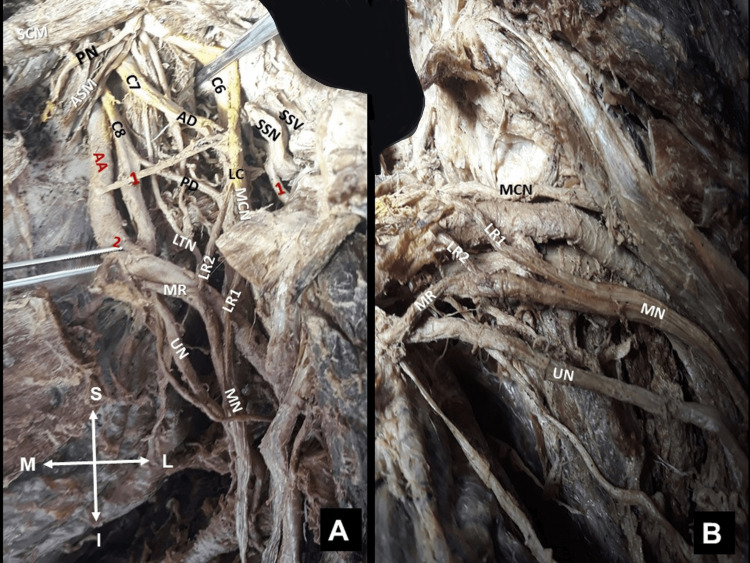
Left axillary view. The atypical formation of the left-sided median nerve (MN) from two lateral roots (LR1 and LR2) and a medial nerve root (MR). (A) Relationships of the aberrant suprascapular artery (2) of origin from the axillary artery (AA), phrenic nerve (PN), anterior scalene muscle (ASM), long thoracic nerve (LTN), sternocleidomastoid muscle (SCM), aberrant suprascapular artery (1), (2) thoracoacromial artery, suprascapular vein (SSV), and (B) suprascapular nerve (SSN) coursing under the superior transverse scapular ligament, ulnar nerve (UN), lateral roots of the MN (LR1, LR2), anterior division (AD), posterior division (PD), musculocutaneous nerve (MCN), and lateral cord (LC). Figures’ orientation (M: medial, L: lateral, S: superior, and I: inferior).

Ipsilaterally, a median nerve with three (two lateral and a medial) roots was found (Figures 5A, 5B). The dissected cadaver was donated to the Anatomy Department, through the “Body Donation Program” after written informed consent.

## Discussion

In the current report, multiple neurovascular variants were unilaterally identified in a dissected formalin-embalmed female donated cadaver. The aberrant SSA (of axillary origin) and atypical course and termination coexisted with 1. an SSN of atypical (low) origin, 2. an atypical course of the LTN components, 3. an atypical formation of the phrenic nerve (by the contribution of multiple roots of the cervical spinal nerves C3-C6), 4. interconnection of the phrenic nerve with the LTN, and 5. an atypical formation of the median nerve (by three roots). Piagkou et al. [2] identified a coexistence of a similar aberrant left-sided SSA (of axillary origin) and its atypical course (between the medial and lateral cords of the BP) with several neurovascular variants, such as a duplication of the musculocutaneous nerve, a multiplication of the lateral thoracic artery, and a subscapular trunk formation. Naidoo et al. [3] recorded a quite low incidence of SSA of typical origin (7%), giving emphasis to the aberrant origin, from the axillary artery’s second part (5%), from the subclavian artery’s third part (4%), from the axillary artery’s first part (2%), and from the subscapular artery (2%). In regards to the alterations in the SSA topography (relationship with the SSN and SSV in the area of the superior transverse scapular ligament), the current report pointed out the course of the suprascapular neurovascular bundle beneath the ligament. This discovery has been classified as type III with frequencies of 10.9% and 12.3% by Polguj et al. [4] and Yang et al. [5], respectively.

The identification of the vascular variants in the anterior neck and supraclavicular area and possible covariants is surgically essential, as during regional surgery, the axillary artery and related branches’ ligation is essential [6]. In case of an SSA injury, the artery might cause embolic events at the SSN small vessels resulting in neuropathy [7]. The knowledge of the suprascapular structures' topography and their relationships, at the superior transverse scapular ligament, is clinically important, especially during the SSA under the ligament [4]. In the current case, the SSN originated from the upper trunk’s posterior division, the so-called SSN low origin. Benes et al. [8] identified a similar variant in 5% of the cases.

The LTN usually arises from the C5, C6, and C7 spinal roots (in 78.1% in Benes et al. meta-analysis) [8], while variant origins have been recorded in 21.9% and classified in types [8]. In the current report, although the LTN originated as typical, an altered course of the C6 anterior ramus through the middle scalene muscle was identified, as well as an interconnection of the LTN with the phrenic nerve, at the level of the thoracoacromial artery origin. Yazar et al. [9] described an unusually similar course of the anterior ramus of the cervical spinal nerves, that pierced the middle scalene muscle, before joining the LTN. In the current case, the piercing of the middle scalene muscle by the anterior ramus from the C6 spinal nerve may cause a partial LTN compression, resulting in reduced severity of the symptoms of pain, paresthesia, and/or paresis [10]. Shilal et al. [11] reported an interconnection of the LTN with the dorsal scapular nerve (originating from the C5 and C6 spinal nerves). Concerning the functional anatomy, the LTN innervates the serratus anterior muscle (a broad and flat muscle that functions as a force pair alongside the trapezius and rhomboid muscles), providing stable anchoring of the scapula to the chest wall. When the serratus anterior does not function properly, it disrupts the coordinated force interaction, leading to medial winging. This dysfunction can arise from either a myopathic or neurogenic cause, with the neurogenic factor being the predominant factor in most instances [12]. LTN palsy may occur due to direct nerve injury (like thoracic trauma or surgery), traction-related damage, or neuralgic amyotrophy, while it has been documented that patients suffering from LTN palsy were more likely to have LTN variants [13].

In the current case, the phrenic nerve was formatted after the fusion (contribution) of the C3, C4, C5, and C6 spinal nerves. This pattern has not been identified in the Mendelsohn et al. [14] study, in which the most common formation pattern was from the C3, and C4 spinal nerves, free of C5 contribution (26%). In this study [14], the phrenic nerve’s typical pattern (after C3, C4, and C5 fusion) was identified in 22%.

In Andrade et al. [15], the C6 contribution was not identified in the formation of the phrenic nerve. Banneheka [16] identified a phrenic nerve receiving contribution from the nerve to the subclavius muscle, the nerve to the sternohyoid muscle, the ansa cervicalis (C1-C3), the BP, the C2 or rarely the C6 spinal nerve (in cases of an accessory phrenic nerve). Moreover, in the current case, the phrenic nerve (branch of the cervical plexus) was interconnected to the LTN (branch of the BP). Goyal and Jain [17] reported an interconnection of the phrenic nerve with the upper trunk of the BP, in its early course in the neck. Although the phrenic nerve has a great clinical interest in the diaphragm supply, the majority of the published studies investigated its anatomy within the thorax [14]. The occurrence of the phrenic nerve variants increases the risk of iatrogenic injury, even in widely performed procedures [18]. Furthermore, the interscalene block of the BP that has been implemented in various orthopedic procedures, including shoulder, clavicle, and humerus surgery could affect both the phrenic and the long thoracic nerves leading to adverse effects. The anterior approach of the BP interscalene block has been associated with a phrenic nerve injury (in up to 8%); hence, the posterior approach is mainly utilized [18,19] which remains risky for the LTN and the dorsal scapular nerve injury [19]. To avoid such complications, meticulous anatomical knowledge of this area is of utmost importance, and the use of ultrasound guidance and nerve stimulator during the BP is also essential. Moreover, the interconnection of the phrenic nerve with other nerves of the adjacent area, in a possible injury of the phrenic nerve or the adjacent interconnected nerve, could affect the other interconnected nerve, causing partial paralysis.

Variations in the origin of these nerves and further communications are important to clinicians accessing the axillary and supraclavicular regions. This knowledge is essential in reconstructive procedures for various types of BP injuries, surgeries for neck tumors, or BP blocks [20].

## Conclusions

The current report highlights the coexistence of an aberrant SSA (of axillary origin) with other multiple neural variants of the adjacent area (cervical and brachial plexus branches). It also emphasizes the aberrant SSA’s variant course and termination under the superior transverse scapular ligament and variant relationship with the SSV and SSN. The report recorded the atypical origin of the phrenic nerve, the atypical passage of the anterior ramus of the C6 spinal nerve (through the middle scalene muscle) before the LTN formation, the interconnection of the phrenic nerve with the LTN, and the atypical formation of the median nerve. Thus, it highlights the value of the detailed step-by-step dissection in identifying all possible covariants. Knowledge of such neurovascular variants is of paramount value for surgeons operating in the supra- and infraclavicular areas. The identification of the variable origin of the nerves of the BP supraclavicular part may help to reduce the rate of iatrogenic injury during axillary interventions. Efforts should be made to expand the number of cadaveric studies investigating the origin, course, interconnection, and branching pattern of these nerves and their related covariants, thus unifying them in a systematic way their study.

## References

[1] Tountas CP, Bergman RA (1993). Anatomic Variations of the Upper Extremity.

[2] Piagkou M, Tsakotos G, Chytas D (2023). An unusual combination of a bilateral aberrant suprascapular artery with neurovascular structures variants. Surg Radiol Anat.

[3] Naidoo N, Lazarus L, De Gama BZ, Satyapal KS (2014). The variant course of the suprascapular artery. Folia Morphol.

[4] Polguj M, Rożniecki J, Sibiński M, Grzegorzewski A, Majos A, Topol M (2014). The variable morphology of suprascapular nerve and vessels at suprascapular notch: a proposal for classification and its potential clinical implications. Knee Surg Sports Traumatol Arthrosc.

[5] Yang HJ, Gil YC, Jin JD, Ahn SV, Lee HY (2012). Topographical anatomy of the suprascapular nerve and vessels at the suprascapular notch. Clin Anat.

[6] Lamb DD (2010). Radical neck dissection. Surg Technol.

[7] Ferreira H (2015). Variations in patterns of branching of the thyrocervical trunk. Int J Pharm BioSci.

[8] Benes M, Kachlik D, Belbl M (2021). A meta-analysis on the anatomical variability of the brachial plexus: Part II: Branching of the supraclavicular part. Ann Anat.

[9] Yazar F, Kilic C, Acar HI, Candir N, Comert A (2009). The long thoracic nerve: Its origin, branches, and relationship to the middle scalene muscle. Clin Anat.

[10] Williams AA, Smith HF (2020). Anatomical entrapment of the dorsal scapular and long thoracic nerves, secondary to brachial plexus piercing variation. Anat Sci Int.

[11] Shilal P, Sarda RK, Chhetri K, Lama P, Tamang BK (2015). Aberrant dual origin of the dorsal scapular nerve and its communication with long thoracic nerve: an unusual variation of the brachial plexus. J Clin Diagn Res.

[12] Wu F, Ng CY (2023). Long thoracic nerve palsy: when is decompression indicated. J Hand Surg Glob Online.

[13] Ormsby NM, Hawkes DH, Ng CY (2022). Variation of surgical anatomy of the thoracic portion of the long thoracic nerve. Clin Anat.

[14] Mendelsohn AH, DeConde A, Lambert HW (2011). Cervical variations of the phrenic nerve. Laryngoscope.

[15] Andrade LS, Jaison J, Jyotsna B, Lewis CR, Prasanna LC (2021). A study on the variants of phrenic nerve roots with histological correlation. Transl Res Anat.

[16] Banneheka S (2008). Morphological study of the ansa cervicalis and the phrenic nerve. Anat Sci Int.

[17] Goyal N, Jain A (2019). Variant communication of phrenic nerve in neck. Surg Radiol Anat.

[18] Prates Júnior A, Vasques LC, Bordoni LS (2015). Anatomical variations of the phrenic nerve: an actualized review. J Morphol Sci.

[19] Mian A, Chaudhry I, Huang R, Rizk E, Tubbs RS, Loukas M (2014). Brachial plexus anesthesia: a review of the relevant anatomy, complications, and anatomical variations. Clin Anat.

[20] Feigl GC, Litz RJ, Marhofer P (2020). Anatomy of the brachial plexus and its implications for daily clinical practice: regional anesthesia is applied anatomy. Reg Anesth Pain Med.

